# Who is Margarita? The legacy of the doctor who became a writer

**DOI:** 10.15694/mep.2018.0000189.1

**Published:** 2018-09-04

**Authors:** Artur Sharluyan

**Affiliations:** 1 Son Espases Universitary Hospital

**Keywords:** Medicine and literature, doctors as writers, heart and soul.

## Abstract

This article was migrated. The article was marked as recommended.

Literature offers an exceptional means to transmit education in values and enhance critical thinking. Mikhail Bulgakov started his life as a doctor and left us a legacy as a master of writers. His book “The Master and Margarita” continues to generate innumerable debates and interpretations about its meaning. Through fantastic world Bulgakov links the ancient roots of Christian culture with soviet totalitarian and atheistic life, raising universal questions about mortality, universal ethical dilemmas, and the very existence and meaning of the soul. For medical trainees it is important to at least consider their position on these important issues. This short review of the book offers some keys to understand the novel under a new perspective and help understand its symbolism.

## Mikhail Bulgakov

**Figure:**
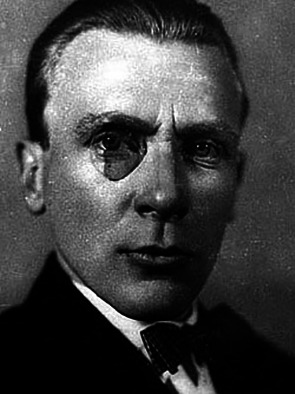


Mikhail Bulgakov, one of the most acclaimed Russian writers of the twentieth century, was born in 1891 in Kiev ( *Russian Empire).* In 1916 he graduated from the Medical Faculty of the St.Vladimir University in Kiev. During the First World War he volunteered with the Red Cross. Being badly injured in the front he injected him with morphine to palliate chronic pain and became an addict for next years. During the Revolution and Civil War he was drafted as an army physician and barely survived after typhus in 1919. This violent career deeply influenced his life. Some of his experiences are portrayed in the “ *Stories of a young doctor*” and in the magnificent “ *Morphine*”. The novel “ *The Master and Margarita*”, considered his work of mayor transcendence is the object of this brief article. The novel, banned for obvious reasons in a repressive and declared atheist state (USSR), was secretly distributed in the form of manuscripts in the late 60s. The story continues to generate innumerable debates and interpretations about its meaning.

## Briefly about the novel and characters

The story intertwines 3 arguments. In the first the **Devil** ( **Woland a magician**) and his retinue arrive in Moscow, altering the imposed normality of life and literary circles. The second is about **the master, a writer**, who writes a novel about **Pontius Pilate** and his act of cowardice in ancient Jerusalem, resulting in **Jeshuas** death (Jesus Crists historical portrait.. or maybe not). For this writing the Master was rejected and repudiated and he burnt the manuscript, (a clear autobiographic element). The third **Margarita** and master romance, a story of true love and beauty. Margarita agrees to play a role as a Queen, at the Great Ball at Satana´s to have a chance to meet again her beloved Master. The 3 stories different in style and form, are interlaced through the encounters between their main characters, their decisions and both in form and in content embrace everything excluded from Soviet ideology and its literature.

**Ivan Homless** is a poet, listener, witness and the link of all 3 stories, just like us, who are viewers who try to discover and understand something.

**The narrative voice**, always the same, using irony, language of parable, parody, and changing from narrative prose to lyrical poetry unites the whole and allows Bulgakov to exploit the theatricality of the scenes. (Richard Pavear analyses the structure and content of the novel in an acute and accurate way in his introduction to the Penguin books edition of 2017).

Fantastic, everyday or grotesque characters come into life from the author’s profound ideology of good and evil seen from the perspective of one’s own conscience, the belief in the prevalence of deep spiritual and moral values over selfishness and materialism. The most praiseworthy ideals, which otherwise would seem utopian, become human and tangible thanks to the sense of humor that accompanies Woland, embodied Satan who nevertheless only touches and punishes those who deserve it.

## Interpretations

The novel can be understood on many levels. It is both socio-political satire of the modern world of his time, a deep philosophical reflection on what it means to be human, and a unique and original vision about the meaning of the “soul”. The fact that Bulgakov wrote several versions and burned the original manuscript (just like his character) during a time of political, physical and moral repression does not help to reduce the complexity of the book.

The novel is extremely rich in symbology, allegory, satire and fantasy, which, apart from creating a unique atmosphere, predisposes to multiple interpretations. This happens with the usual explanations, which focus on the first part of the book and its allusion to the atheistic propaganda of Soviet literature, devoid of genuine creativity in the years of terror of the 20s. The allegory of the Devil visiting Moscow in those times is easy to understand. Some attempt to see a real people in the characters such as Bulgakov’s 2nd wife Elena Sergeevna in Margarita, Lenin or Stalin in Woland, writers like M. Gorki in Master and so on, do not have any sense at all. Abstract, changing, fantastic characters and their relations, rather than to be or not to be something or someone concrete, help us to feel the complexity of human being.

## Who is Margarita?

Actually, Bulgakov himself handles this question to us. Ivan, a listener of the story asks Master: “Who is She?” (Chapter 13) but does not receive a response. I am sure that Bulgakov purposefully invites us to give the answer, as in the theatre where the spectator participates in the action, actively taking with him new thoughts.

Bulgakov was a screenwriter and reading the novel gives the impression that only 5 above mentioned characters are actors on the stage, while other things (like Margarita’s marriage, her former life, sceptic Berlios, the events in Griboyedov, the Great Ball at Satana´s..) are only a scene. Despite Margarita is recognized to stand for love, beauty, true, and courage, her story is used to be interpreted as a romance. I suggest that with an unprecedented resource in literature, Bulgakov personifies master´s Soul in Margarita.

Master, when we know him, is admitted to a psychiatric clinic. The reader should know that in Russian for a long time, psychiatric patients were called “Душевнобольной” “sick of the soul”. This is how the author tells us directly that Master, is lost and sick because he has “lost” his soul (Chapter 13), and Margarita is who performs all those actions of which he would be a participant and author. Bolgakov insists with the almost only words Margarita has written to say goodbay: (Chapter 20) “I´ll go, but you should know that you are a cruel man! They´ve devastated your soul!” She means if he decides to live without her, without his soul, betraying his values.

Although some part of our being belong to eternity, Bulgakov presents the idea of the soul not as a something abstract existent after death, but as an action expressed in the matter of living, and specifically embodying compassion, mercy, forgiveness, and love.

A different vision of the soul.

In chapter 21(“Invisible and free!”), Margarita becomes a witch after smearing herself with Azasello’s (demon´s) cream. This is how Margarita feels, her real state. Invisible and free soul of the poet. Margarita represents the freedom of the artist in unfree world. One sense of art is precisely breaking with the established canons, offering a new and different vision, manifesting through creativity the search for beauty and truth. Thus the first qualities of Margarita: “Invisible and free!” manifest the essential characteristics of the “artist’s “soul”, Masters soul.

Margarita “..who was not in need of money and happily married..” owning everything she wanted, a woman who becomes Masters lover, writes about herself: “I became a witch from grief and calamities that have struck me” (Chapter 20). Material welfare and security, does not guarantee peace of mind and happiness or spiritual well being.

Now I briefly quote the places in the book where Margarita’s actions directly manifest this idea.

Love (Chapter 19, 21). Margarita embodies the eternal Love. Love soaks all the actions that Margaret performs and gives meaning to her existence. Love for the master, for life, for those who suffer.. Even anger ends with an act of appeasing a frightened child in chapter 21.

Mercy (Chapter 24). With “only one desire to ask” in return for helping Satan, Margot asks “mercy for Frieda”, the woman who committed one of the most terrible crimes killing her baby.

Compassion and Forgiveness (Chapter 32). When overcoming the barriers of time, they meet Pilate and their destiny is about to be decided: “Let him go! Piercingly cries Margo”, after “..her perfectly calm face clouded over with compassion”. Observe that she is who is taking the action, although all included Master, are present. Only the last action is accomplished by Master who absolves Pontius Pilate, but only while being in eternal union with Margarita.

Finally, the master is the one who “has the keys” (Chapter 13) that allow him to enter our isolation cells, to bring some spiritual light to the loneliness of the sick witness, like Ivan or maybe a reader. In the end, with the physical death of Master and Margarita, Bulgakov emphasizes that everything happens in the spiritual world. I will not comment on the end so as not to spoil it to the readers for whom the book is yet to be discovered. But the last pages, full of poetry, transport the reader to a powerful world of feelings of the end of life, where the soul, having seen everything and suffered, calls for calm and peace of rest.

I think Bulgakov deliberately disguised writing about the soul as a love story, in times when the very existence of soul and spirit was denied. Even if you do not accept the idea of Margarita as masters soul and not a person, she still the core character of the novel who transmits all that feeling of beauty, compassion, love, courage and at last meaning. Definitely she is a soul and spirit of the entire novel, expressing human heart and soul.

I understand that it is difficult to capture the nuances for those who have not read the novel, and I intend to encourage the reader interested in literature to know this magnificent and profound work on the spiritual world of the human being. In times of iniquity, master Bulgakov has written about the most important.

## Take Home Messages

• Literature offers an exceptional means to transmit education in values and enhance critical thinking.
• Russian writer M. Bulgakov started his career like a doctor but left a legacy as a briliant writer.

## Notes On Contributors

Dr. A. Sharluyan, was born in Armenia (USSR) and lives in Spain working as an Pediatric Intensivist dedicated to the transport of critical patients.
