# Supplementary material for: Who is Margarita? The legacy of the doctor who became a writer
Source: MedEdPublish (2016). 2018 Sep 4;7:189. [Version 1] doi: 10.15694/mep.2018.0000189.1 (PMC10701828; doi:10.15694/mep.2018.0000189.1)
Supplement: Peer review history

# Reviewer report: Reviewer response for version 1

**DOI:** 10.21956/mep.19620.r29178

**Published:** 2018-09-09

**Authors:** Irina Markovina

**Affiliations:** 1 Sechenov First Moscow State Medical University (Sechenov University)

This review has been migrated. The reviewer awarded 3 stars out of 5 To me as a reviewer this article of the section Personal view and opinion seems interesting for a number of reasons. I believe it may be interesting for the themed issue readers for the same reasons. First, the author who is a medical doctor analyses one of the most outstanding literary work of the past century written in Russian. Second, the writer whose book is analyzed, Mikhail Bulgakov, was a medical doctor at the beginning of his career. Third, the article definitely broadens the context for discussion of the Humanities role in medical education and medical profession in general.I will comment on the third point first. The attempt of a doctor to analyze the novel, which deals with the most fundamental questions of human existence, deserves appreciation. The ideas of the article’s author is an excellent example of how doctors can use literature to keep their minds and souls healthy, to preserve their spiritual wellbeing, so to speak. The very fact that the analyst is an amateur shows how high quality literature can influence a doctor’s mind. In my opinion, it is a convincing example of how humanities “work” in a real life of a doctor. This analytical activity, the ability to appreciate literature definitely give him a lot of support both to his mind and to soul. My comment on the second point aims to criticize the author for not checking the facts of Mikhail Bulgakov’s life before writing the article. In fact, Elena was his third wife not the second one as mentioned in the article. As for point 1 of my comment, it is important to emphasize again that the article is an excellent example of a dialogue between two doctors, the one living in the XXI century trying to decode the writer’s interpretation of the eternal questions. In conclusion, the article may be regarded as a good illustration to another piece published here, namely What’s a book club doing at a medical conference by M. Chisolm et al.

Reviewer Expertise:

NA

No decision status is available

# Reviewer report: Reviewer response for version 1

**DOI:** 10.21956/mep.19620.r29180

**Published:** 2018-09-04

**Authors:** Benjamin Burrows

**Affiliations:** 1 NHS

This review has been migrated. The reviewer awarded 2 stars out of 5 I agree with the author that there is a rich repository of material in fiction to assist with the teaching of the medical humanities. I also believe that authors from diverse sociopolitical backgrounds are an especially rich source of material for this. They enable us to develop our appreciation of the great diversity of experience, something essential for helping us to understand our patients, many of whom have completely different life experiences to our own.However I am not sure I have any greater understanding of the authors work or symbolism from this article. I appreciate the difficulty in producing a review of a book many people are ignorant/unaware of, however I feel a little more work would provide many of us with a greater understanding of the author and their potential as a contributor to the teaching of medical humanities to future doctors.

Reviewer Expertise:

NA

No decision status is available

# Reviewer report: Reviewer response for version 1

**DOI:** 10.21956/mep.19620.r29177

**Published:** 2018-09-04

**Authors:** P Ravi Shankar

**Affiliations:** 1 American International Medical University

This review has been migrated. The reviewer awarded 2 stars out of 5 Thank you for the opportunity to review this interesting book review. The novel seems interesting but as I have not yet read the novel I found some of the description difficult to follow. The author should concentrate on the relevance of the novel to medical students and for medical humanities modules. What are the specific benefits for medical students which could be derived from the book? How can the novel contribute to a medical humanities module? What topics can be examined through the novel?

Reviewer Expertise:

NA

No decision status is available

# Reviewer report: Reviewer response for version 1

**DOI:** 10.21956/mep.19620.r29181

**Published:** 2018-09-04

**Authors:** Joan B Soriano

**Affiliations:** 1 Hospital La Princesa

This review has been migrated. The reviewer awarded 4 stars out of 5 This text is an interesting book review on Bulgakov’s book “Master and Margarita?”. It compels you to read this novel by an author who was a medical doctor who lived (and suffered) war and repression. It is a concise manuscript and it reads very well. However, the book review is complex, likely as the novel itself, full of symbols and with many characters.On the title’s question, perhaps I am missing that in the novel, Margarita is a 30-yr old housewife, and married to a rich, famous military engineer she doesn't love and with whom she has no children. She lives in a large Moscow apartment. She falls in love with a mysterious writer, who she called Master. Her feelings of love, despair, mercy, compassion and forgiveness are nicely presented in this book review. These feelings must be common to both women (and men) who accept the challenge to abandon an established relationship and following someone else passionately, by definition an irrational act.So many things to read, so little time…. If only, perhaps I am missing the rationale, just hinted in the abstract, on why reading this novel is recommended for medical trainees: “…raising universal questions about mortality, universal ethical dilemmas, and the very existence and meaning of the soul.”

Reviewer Expertise:

NA

No decision status is available

# Reviewer report: Reviewer response for version 1

**DOI:** 10.21956/mep.19620.r29179

**Published:** 2018-09-04

**Authors:** Trevor Gibbs

**Affiliations:** 1 AMEE

This review has been migrated. The reviewer awarded 1 stars out of 5 I am sure that this paper represents a very good treatise on the writing of Bulgakov, but I personally found it very difficult to link these words into the education of a healthcare professional. I am sure that both of the author's take Home messages are equally true but again, their relationship to education is lacking.At a time when the Humanities is trying to find its rightful place in medical education, it is important to be as clear as possible in linking the two. I think that this paper doesn't make that connection.

Reviewer Expertise:

NA

No decision status is available
